# Academic transition shock: How first-year public health students navigate unfamiliar learning demands in computer science education

**DOI:** 10.3389/fpsyg.2026.1825898

**Published:** 2026-08-04

**Authors:** Omar Alobud

**Affiliations:** King Saud bin Abdulaziz University for Health Sciences, King Abdullah International Medical Research Center and Ministry of National Guard—Health Affairs, Riyadh, Saudi Arabia

**Keywords:** academic transition, computer science education, learning strategies, public health education, qualitative research

## Abstract

**Background:**

Public health students often enter training with learning strategies optimized for memorization and recall that prove inadequate when confronted with unfamiliar cognitive demands. This study explored how first-year public health students experience and navigate academic transition when encountering an introductory computer science course that challenges their established learning approaches.

**Methods:**

A qualitative interview study was conducted with 10 first-year public health students enrolled in a mandatory computer science course. Semi-structured interviews explored students' expectations, experiences of difficulty, emotional responses, and adaptive strategies. Data were analyzed using reflexive thematic analysis.

**Results:**

Four interconnected themes emerged: expectation shock and the collapse of familiar patterns, encountering unfamiliar dimensions of difficulty, developing adaptive responses through trial and necessity, and reframing academic competence from recall to reasoning. Students initially relied on memorization-based strategies that had proven successful in prior education, but programming tasks exposed the inadequacy of these approaches. Through iterative engagement with unfamiliar demands, students gradually developed reasoning-oriented strategies, increased tolerance for ambiguity, and reconceptualized learning as an iterative process rather than accurate reproduction of information.

**Conclusions:**

Academic transitions that challenge students' implicit learning assumptions can serve important developmental functions by exposing limitations in established approaches and creating conditions where cognitive adaptation becomes necessary. Public health curricula may benefit from intentionally incorporating early learning experiences that resist memorization-based strategies, helping students develop adaptive learning capabilities before encountering high-stakes fieldwork and practice contexts where flexible problem-solving is essential.

## Introduction

Public health students consistently report elevated levels of psychological distress during their training, with anxiety and stress documented across diverse educational settings and stages of professional development. Research demonstrates that substantial proportions of health professions and graduate public health students experience clinically significant anxiety symptoms, often manifesting as worry about academic performance, difficulty managing competing demands, and concerns about their capability to meet the expectations placed upon them (Juul et al., 2022; Kjær et al., 2022; Quek et al., 2019; Agroia et al., 2024). While some of this distress can be attributed to the inherently demanding nature of public health education, evidence suggests that stress intensifies particularly during periods of transition, when students must navigate shifts in learning environments, assessment methods, or the fundamental nature of what they are being asked to learn (Sinval et al., 2025; Wolf, 1994). These transition points appear to function as vulnerability windows where pre-existing anxieties become amplified and where students' capacity to cope is tested most directly. Understanding what makes educational transitions particularly stressful, and how students navigate these challenges, is therefore crucial for designing curricula that support student wellbeing while maintaining academic rigor and preparing students for the realities of public health practice.

A growing body of literature suggests that much of the difficulty students experience during educational transitions stems not simply from increased workload or more challenging content, but from fundamental misalignments between how students have learned to learn in previous contexts and what new learning environments demand of them. Many students enter public health programs having succeeded primarily through strategies that emphasize memorization, careful note-taking, and reproduction of information presented by instructors, approaches that prove highly effective in educational systems structured around lectures and examinations that reward recall of epidemiological concepts, health policy frameworks, and biostatistical procedures (Dunham et al., 2017; Surmon et al., 2016). However, these students often report feeling unprepared when they encounter learning contexts that require different cognitive approaches, such as synthesizing evidence across heterogeneous datasets, reasoning through ambiguous population health scenarios, or applying analytical principles to novel public health problems. This sense of unpreparedness reflects not a lack of knowledge or intellectual capability but rather a gap between the learning strategies students possess and the strategies demanded by their new environment. The learning environment itself plays a powerful role in shaping students' experiences, with research demonstrating that students' perceptions of difficulty, their emotional responses to challenge, and their ultimate success are all substantially influenced by whether the environment's demands align with their existing competencies and expectations (Louey et al., 2025; Zamora et al., 2023; Lim et al., 2024b). When students encounter environments where familiar approaches no longer work as expected, they experience a particular form of stress that combines cognitive confusion about what to do differently with emotional distress about whether they are capable of adapting successfully.

Educational theory provides useful frameworks for understanding why certain learning strategies fail to transfer across contexts and how students adapt when confronted with these failures. Research on transfer of learning demonstrates that knowledge and skills acquired in one context often do not spontaneously apply to new situations, even when those situations appear superficially similar, because effective performance depends not just on possessing knowledge but on recognizing when and how that knowledge is relevant (Peters et al., 2018; Rohani et al., 2024). When transfer breaks down—when students attempt to apply familiar strategies and find them inadequate—the resulting difficulty creates both a problem and an opportunity. The problem is immediate: students must complete tasks without access to their usual approaches, creating frustration and uncertainty. The opportunity, however, is that these moments of breakdown make learning processes visible and subject to conscious examination in ways that automatic, successful performance does not. Students begin to notice what they are doing, why it is not working, and what might need to change. This kind of learning through experience, where understanding emerges from active engagement with authentic tasks rather than from explicit instruction, has been identified as particularly powerful for developing adaptive expertise that can be applied flexibly across diverse situations (Lee et al., 2024; Dornan et al., 2014; Lave and Wenger, 1991). The challenge lies in creating educational experiences where transfer failures occur in contexts supportive enough that students can work through them productively rather than becoming overwhelmed or disengaged. To make the study's theoretical commitments explicit, this research is situated within two complementary frameworks. The first is situated cognition, which holds that knowing and learning are inseparable from the authentic activity and context in which they occur, so that competence develops through participation in genuine practice—here, the practice of writing and debugging code—rather than through the acquisition of decontextualised facts (Lave and Wenger, 1991). The second is transfer of learning, which concerns whether and how strategies acquired in one setting are mobilized in another. Read together, these lenses frame programming as learning-by-doing in which established, memorization-oriented strategies are expected to transfer poorly, and they guided both the design of the study—selecting an authentic, practice-based course as the site of enquiry—and the analysis, sensitizing interpretation to how students participated in, and made sense of, unfamiliar practice.

Computer science education, particularly for students with no prior programming background, offers a distinctive context for observing how public health students respond to academic transitions that challenge their established learning approaches. Programming requires logical reasoning, systematic problem decomposition, debugging through iteration, and tolerance for ambiguity—cognitive demands that differ substantially from the memorization and recall strategies that dominate much pre-public health education. When public health students encounter programming tasks, many find that carefully studying examples and attempting to memorize syntax does not translate into the ability to write functioning code, because programming tends to reward understanding of underlying logic and principles more than the reproduction of memorized patterns (Luxton-Reilly et al., 2018; Lai and Chen, 2022; Farooq and Anwar, 2024; Hao et al., 2022). This creates conditions where students experience what might be termed academic transition shock—a collision between their expectations about how learning should work and the reality of what a new domain actually demands. Such experiences can function as stressors that hinder learning if students become discouraged and disengaged, but they can also challenge students in productive ways that stimulate adaptation and the development of new strategies (Rudland et al., 2022). The nature of students' responses to this transition, the specific difficulties they encounter, and the strategies they develop for managing unfamiliar learning demands remain incompletely understood, particularly in the context of public health education where most research focuses on fieldwork and practicum transitions rather than academic transitions within coursework (Adnan et al., 2024; Surmon et al., 2016). The present study therefore aims to explore how first-year public health students experience the academic transition into a computer science course, examining the specific challenges that arise when familiar learning strategies prove inadequate and the adaptive responses students develop as they navigate this unfamiliar learning environment. Guided by these aims and by the situated cognition and transfer-of-learning frameworks outlined above, the study addressed three research questions: (1) How do first-year public health students experience the academic transition into an introductory computer science course? (2) What difficulties arise when their established, memorisation-oriented learning strategies fail to transfer to programming tasks? (3) What adaptive strategies do students develop as they navigate these unfamiliar learning demands?

## Method

This study employed a qualitative research design to explore how first-year public health students experience the academic transition into a computer science course and how they respond when familiar learning strategies prove inadequate in this new context. These dimensions of experience cannot be adequately captured through quantitative measures of performance or stress levels, as they require detailed exploration of how students perceive and make sense of their learning processes in their own terms.

Ten first-year public health students enrolled in a mandatory introductory computer science course participated in this study. All participants were in their first semester of a public health degree program and had no formal background in computer science or programming, ensuring that they were genuinely encountering unfamiliar learning demands rather than applying prior expertise. Participants were recruited through announcements in the course, with purposive selection used to ensure diversity in prior academic backgrounds, initial confidence levels about the course, and self-reported learning preferences. None of the participants had withdrawn from the course or expressed intentions to do so, meaning the sample captured students who were actively working through the transition rather than those who had disengaged entirely (Malterud et al., 2016). The course was a compulsory component of an undergraduate public health degree and was taken by the entire first-year cohort of students; the 10 interviewees therefore constituted a purposive sub-sample of this cohort rather than a self-standing enrolment.

The course was delivered over one semester and combined weekly lectures introducing programming concepts with smaller hands-on sessions in which students wrote and debugged code, supported by regular formative programming assignments and at least one summative assessment. Instruction therefore emphasized active, problem-based practice and iterative feedback rather than didactic transmission alone, an approach oriented toward developing logical reasoning and problem-solving.

Data were collected through individual semi-structured interviews conducted approximately midway through the computer science course, after students had completed several programming assignments and experienced at least one assessment. This timing was deliberate, allowing participants sufficient exposure to the course's demands to have encountered difficulties and begun developing responses, while ensuring that their experiences remained vivid and accessible rather than reconstructed from distant memory. Interviews lasted between 40 and 70 min and took place in private settings where participants could speak candidly without concern about being overheard by instructors or peers. The interview guide opened with broad invitations for participants to describe their initial expectations about the course and how their actual experiences compared to those expectations, then moved to more focused exploration of specific moments when they felt confused, frustrated, or surprised by what the course demanded (O'Reilly, 2012). Follow-up questions probed how participants responded when their usual study strategies did not seem to work, what they found most difficult about learning programming compared to other subjects, and whether they noticed any changes in how they approached learning as the course progressed. Participants were also asked to reflect on how the computer science course compared to their other public health courses and whether the challenges they encountered felt qualitatively different from typical academic difficulty. All interviews were audio-recorded with explicit permission and transcribed verbatim to preserve participants' exact language, as subtle differences in how students described their experiences often revealed important nuances in their perceptions of difficulty and adaptation. All interviews were conducted by an independent researcher unconnected to teaching or assessment. This distinction matters because the interviews took place mid-course: to reduce the risk that students would tailor their responses to an assessor, the interviewer held no role in grading or in decisions affecting participants' progression, participation was voluntary and confidential, and students were assured that their responses would not be shared with teaching staff or affect their standing in the course. The study was conducted with ethical approval, and all participants gave informed consent to take part and to be audio-recorded.

**Figure 1:**
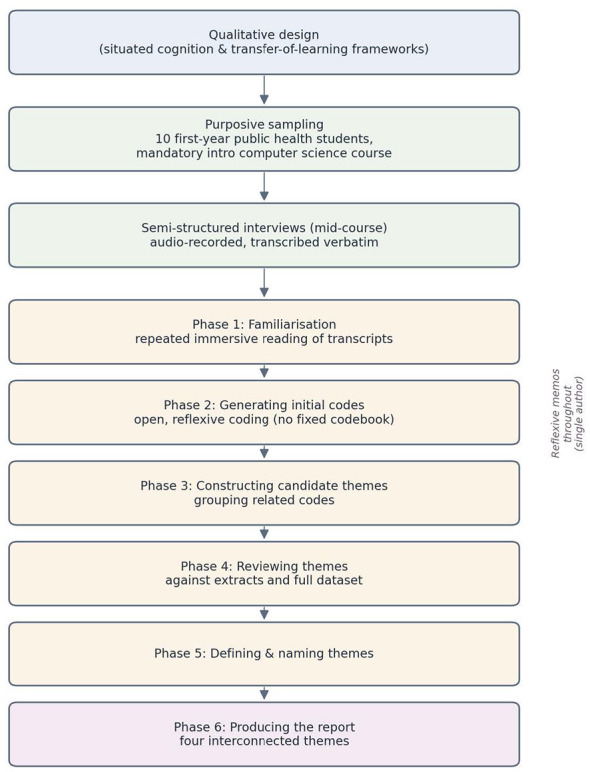
Overview of the study's qualitative design and the six-phase reflexive thematic analysis process, from familiarization to the final four themes.

Interview transcripts were analyzed using reflexive thematicanalysis, an approach that emphasizes the researcher's interpretive role in constructing themes from the data while remaining rigorously grounded in what participants actually expressed (Braun and Clarke, 2021). This method was selected for its flexibility in addressing research questions that focus on experience and meaning-making, and for its explicit recognition that analysis involves interpretive choices shaped by theoretical frameworks and research aims rather than mechanical extraction of pre-existing patterns. Analysis proceeded through multiple iterative phases, beginning with immersive, repeated reading of all transcripts to develop comprehensive familiarity with the dataset as a whole. Initial codes were then systematically generated to capture specific features of the data relevant to the research questions, such as descriptions of unmet expectations, moments when familiar strategies failed, emotional responses to difficulty, and accounts of trying new approaches. These codes were applied across the entire dataset with attention to both commonalities and variations in how different participants described similar experiences. Coded segments were then organized into broader candidate themes by identifying patterns that connected related codes and reflected overarching ideas about how students experienced and navigated the academic transition. Analysis then continued through the remaining phases of Braun and Clarke's approach: candidate themes were reviewed against both the coded extracts and the full dataset to confirm that they were coherent, distinct, and well-evidenced; themes were then defined and named to capture the essence of each; and the final phase involved selecting illustrative data extracts and producing the analytic narrative reported below. Consistent with the reflexive tradition, coding was open and organic rather than governed by a pre-specified codebook or fixed coding frame, and inter-rater reliability was not sought, because reliability metrics presuppose that themes exist independently of the analyst and can be reliably re-identified—an assumption at odds with the reflexive premise that theme development is an interpretive act shaped by the researcher's theoretical position. Reflexive thematic analysis was preferred over a purely emergent or grounded-theory-style coding process precisely because the study was theoretically informed: rather than claiming to derive themes from a blank-slate reading of the data, the analysis openly drew on the situated cognition and transfer-of-learning frameworks as sensitizing concepts while remaining grounded in participants' own accounts. Throughout, the author kept reflexive memos to make interpretive choices visible and to interrogate how prior assumptions might shape the developing themes. The full interview guide is provided in Appendix A, and the overall analytic process is summarized in Figure 1. As the sole researcher, the author's own disciplinary background of computer education shaped these interpretive choices, and this positionality was kept in view throughout coding.

## Results

The analysis revealed four interconnected themes that capture how first-year public health students experienced the academic transition into the computer science course and how they responded when familiar learning strategies proved inadequate. These themes illustrate a trajectory from initial expectations shaped by prior educational success, through confrontation with unfamiliar forms of difficulty that resisted familiar approaches, to gradual development of adaptive responses that often involved reconceptualizing what learning means and what counts as academic competence. Together, these themes depict academic transition not merely as an increase in difficulty but as a fundamental shift in the nature of learning demands that requires both cognitive and emotional adaptation. Table 1 summarizes these four themes, the key challenges that shaped each, and the links between them.

**Table 1:** The four interconnected themes, their key contributors or challenges, and the links between them that create opportunities for student adaptation and curricular reform.

Theme	Key contributors/challenges	Adaptive opportunity and link to next theme
1. Expectation shock and the collapse of familiar patterns	Confidence from prior success; memorization and note-taking assumed to suffice; effort no longer predicting outcome; panic and self-doubt	Makes implicit learning strategies visible—the precondition for recognizing that new demands (Theme 2) require change
2. Encountering unfamiliar dimensions of difficulty	Ambiguous, open-ended tasks; debugging and iteration; logical reasoning; contrast with lookup-based epidemiology	Reveals which specific demands resist old strategies, prompting experimentation (Theme 3)
3. Developing adaptive responses through trial and necessity	Writing code despite uncertainty; using errors as clues; peer sense-making; reframing frustration as normal; process-focused goals	Repeated experimentation accumulates into a changed view of competence (Theme 4)
4. Reframing academic competence from recall to reasoning	Competence redefined as reasoning, tolerance of ambiguity, and persistence; understanding emerging from doing	Feeds back to reshape approaches to difficulty across other courses and future practice

The first theme, expectation shock and the collapse of familiar patterns, emerged from participants' accounts of entering the computer science course with confidence based on their prior academic achievements, only to encounter a learning environment where their established approaches simply did not function as expected. Many students described arriving at the public health program having consistently succeeded through careful attention in lectures, thorough note-taking, and systematic memorization of material, strategies that had reliably produced good grades throughout their previous education. Several participants explained that they expected the computer science course to follow a similar pattern—that if they attended lectures attentively, studied the examples provided, and memorized the syntax and commands demonstrated by instructors, they would be able to complete assignments and perform well on assessments. One student recounted spending an entire evening carefully copying code examples from lecture slides into a notebook and reviewing them repeatedly, fully expecting that this preparation would enable them to tackle the first programming assignment, only to find themselves utterly lost when actually attempting to write code because the assignment did not match any memorized example closely enough. Another participant described the disorientation of realizing that despite having studied diligently and feeling they understood the lecture material, they could not translate that understanding into functioning programs, an experience entirely unlike anything they had encountered in epidemiology or health policy courses where studying reliably led to successful performance. Students articulated a profound sense of shock not just at finding the course difficult, but at discovering that the relationship between effort and outcome had fundamentally changed—that the study strategies they trusted no longer reliably produced results. This collapse of familiar patterns created significant emotional distress, with several participants describing feelings of panic or inadequacy as they questioned whether they simply lacked the intellectual capacity for this type of learning, a particularly threatening interpretation given that academic competence formed a core part of their identity as successful students who had earned admission to a competitive public health program. As one student put it, “I've always been the student who works hard and does well, so I assumed I'd be fine—then I opened the first assignment and just froze, because none of my usual ways of studying were any use.” Another described the same loss of a familiar rhythm: “I kept waiting for the part where you memorize the steps and repeat them, and it never came, and that was when I started to panic.” For several students the shock was as much about identity as difficulty: “For the first time, working harder was not making it click, and I honestly wondered whether I had been fooling myself all along.”

The second theme, encountering unfamiliar dimensions of difficulty, reflects participants' growing recognition that the challenges they faced in the computer science course were qualitatively different from difficulties they had experienced in other academic contexts. Students described struggling not primarily with the volume of material or the pace of instruction, but with more fundamental aspects of how programming tasks were structured and what they demanded cognitively. Many participants articulated difficulty with the ambiguity inherent in programming assignments, where problems were described in general terms without step-by-step procedures to follow, requiring students to figure out their own approaches rather than applying demonstrated methods. One student explained that they felt paralyzed when assignments said something like ‘write a program that calculates' without specifying exactly how to structure the solution, because they were accustomed to following clear procedures where each step was defined. Another participant described the frustration of debugging, where error messages pointed to problems but did not explain solutions, forcing them to iterate through multiple attempts and learn from failures rather than getting things right on the first try by following instructions carefully. Students also noted difficulty with the logical reasoning required for programming, which demanded thinking through conditional relationships and sequential processes in ways that felt different from memorizing disease surveillance criteria or applying established health policy frameworks. Several participants contrasted programming with studying epidemiology, noting that epidemiology involved learning and applying established disease patterns, thresholds, and analytic procedures that could be looked up and verified in guidelines and textbooks, whereas programming required constructing solutions that did not exist until you created them, making the task feel fundamentally more uncertain and open-ended. The difficulty was not simply that programming was hard, but that it was hard in ways students had not encountered before—ways that resisted their established strategies for managing academic challenges and that seemed to require cognitive approaches they had not developed through prior public health coursework. One participant described this unfamiliar difficulty directly: “It wasn't that there was too much to learn. It was that there was no single method to follow—you had to work out the approach yourself, and I'd never really had to do that before.” Another contrasted it with more familiar coursework: “In epidemiology you can look the method up and apply it, but here the answer did not exist until I built it, and that felt completely different.” The demands of debugging drew particular attention: “The error messages were the worst at first—they told me something was wrong but not what, so I had to reason it out instead of just fixing a step.”

The third theme, developing adaptive responses through trial and necessity, captures how students gradually began experimenting with new approaches as they recognized that familiar strategies were insufficient and that they needed to change something if they hoped to succeed in the course. This adaptation was rarely sudden or systematic; instead, it emerged incrementally through a combination of desperation, observation of what seemed to work slightly better, and gradual willingness to try approaches that initially felt uncomfortable or inefficient. Several participants described reluctantly beginning to write code even when uncertain whether it would work, simply because they had spent so long reading and studying without making progress that attempting something felt necessary even if unsuccessful. One student explained that they started deliberately making mistakes just to see what would happen, running incomplete code to observe error messages and using those messages as clues about what to try next, an approach that felt wasteful and inefficient at first but gradually proved more productive than trying to plan perfect solutions before writing anything. Another participant described discovering that talking through problems with classmates, even when neither person knew the answer, often led to insights that studying alone never produced, because verbalizing their thinking revealed assumptions they were making unconsciously. Students also reported adjusting their emotional responses to difficulty, learning to interpret frustration and confusion as normal parts of the learning process rather than as evidence that they were failing or that something was wrong. Several participants noted that they began setting different goals for themselves—shifting from expecting to understand everything before starting an assignment to aiming simply to make some progress, learn from errors, and gradually build solutions through iteration rather than producing correct answers immediately. These adaptive responses were often described as pragmatic accommodations born of necessity rather than principled changes in learning philosophy, yet they represented meaningful shifts in how students engaged with unfamiliar material and managed the emotional challenges of not knowing what to do. As one student explained, “I stopped trying to get it perfect before I started; I'd just write something, run it, and read the error to work out the next step—honestly, the mistakes were how I learned.” Working alongside peers featured strongly in these accounts: “Half of what I figured out came from sitting with two classmates and talking through why the code would not run; none of us knew the answer, but saying it out loud made it click.” For some, the shift was emotional as much as practical: “The turning point was realizing everyone was stuck, not just me, and once it stopped feeling like proof I did not belong, I could actually focus on the problem.”

The fourth theme, reframing academic competence from recall to reasoning, emerged from participants' reflections on how their understanding of what it means to be academically competent began to shift as they worked through the computer science course. Students described gradually recognizing that success in this context did not depend primarily on how much they could remember or how accurately they could reproduce demonstrated procedures, but rather on their capacity to reason through problems, tolerate ambiguity, and persist through multiple failed attempts. This recognition often came as a revelation that challenged long-held assumptions about the relationship between studying, knowledge, and performance. One participant explained that they had always equated being a good student with having the right answers, but programming forced them to realize that sometimes being a good learner means not having answers and being willing to figure things out through experimentation. Another student described recognizing that in programming, understanding emerged from doing rather than preceding it—that you could not fully understand how a loop worked until you had written several loops, debugged them, and observed how they behaved, which felt backwards compared to how they thought learning should proceed. Several participants noted that this shift in perspective affected how they viewed difficulty more broadly, with some beginning to question whether their struggles in the computer science course indicated deficiency or simply reflected that they were learning something genuinely new that required different capabilities. Students also described increased appreciation for the value of reasoning skills and problem-solving processes as opposed to accumulated knowledge, with a few participants noting that this made them wonder whether public health education more broadly might benefit from emphasizing reasoning and analytical problem-solving rather than focusing so heavily on memorizing established frameworks and procedures. This reframing represented not just a change in study tactics but a deeper reconceptualization of what academic competence entails and how learning actually happens, shifts that had implications extending beyond the computer science course to students' broader educational identities and approaches to challenges in other domains. One participant summed up this reframing: “I used to think being good at a subject meant knowing all the answers; now I think it means being able to sit with not knowing and reason your way through it.” Another linked the change directly to their wider studies: “It changed how I study everything—in epidemiology I used to just memorize which test to use, but now I try to understand why you would pick one, because that is what the coding forced me to do.” A third reframed difficulty itself: “Getting stuck stopped feeling like failure; it started to feel like the normal part of figuring something out.”

## Discussion

The findings from this study reveal that academic transitions can function as critical junctures in students' educational development, not simply because they introduce new content or increase workload, but because they expose and challenge fundamental assumptions about what learning is and how it should proceed (Lucey et al., 2018). Students entered the computer science course carrying expectations shaped by years of educational success achieved primarily through memorization and careful reproduction of demonstrated procedures, assumptions so deeply ingrained that many students were not consciously aware they held them until those assumptions proved inadequate. The computer science course created conditions where these implicit expectations collided with a fundamentally different set of learning demands, producing what might be understood as a form of academic culture shock where students found themselves disoriented not by failing to understand specific concepts but by discovering that the entire framework they used for approaching learning did not apply in this new context. This experience of disorientation and the adaptive responses it prompted suggest that well-designed academic transitions may serve important developmental functions, creating opportunities for students to develop more flexible, adaptive approaches to learning that serve them better across the diverse contexts they will encounter throughout public health training and practice.

The acute distress students experienced during this transition appears rooted less in the objective difficulty of programming itself and more in the mismatch between how students expected learning to work and what the course actually demanded of them. Educational research on learning transitions has documented that students often report feeling unprepared when moving into new learning environments, and that this sense of unpreparedness intensifies when the new environment requires different cognitive approaches or values different kinds of competence than students have previously developed (Lee et al., 2024; Dunham et al., 2017; Peters et al., 2018). The computer science course exemplified this dynamic particularly clearly because programming resists memorization-based approaches so directly—no amount of studying examples or memorizing syntax translates automatically into the ability to write functioning code, because programming fundamentally requires logical reasoning, problem decomposition, and iterative refinement rather than recall. When students attempted to apply their familiar strategies and found them ineffective, they experienced not just frustration with a difficult task but a more existential uncertainty about whether they possessed the capabilities needed for success, an interpretation made more threatening by the fact that academic competence formed a core part of their identity as high-achieving students who had earned admission to a competitive public health program. This suggests that the psychological distress commonly reported during educational transitions may often reflect not simply stress from increased demands but rather the deeper anxiety that arises when students' foundational assumptions about their capabilities and about how learning works are called into question (Sinval et al., 2025; Wolf, 1994). Understanding transitions through this lens helps explain why some students experience profound difficulty even with objectively manageable content, and why interventions focused solely on providing additional instruction or reducing workload often fail to address the core source of students' distress. The issue is not that students need more time or clearer explanations, but that they need support in recognizing why their established approaches are failing and in developing alternative strategies for engaging with unfamiliar learning demands (Rudland et al., 2022).

The adaptive responses students developed emerged not from explicit instruction about how to approach programming differently but through iterative experience with tasks that resisted their familiar strategies, suggesting that learning how to learn often requires direct engagement with challenging experiences rather than advance preparation. Educational theory emphasizing experience-based learning has long argued that meaningful adaptation occurs when learners engage with authentic tasks, encounter difficulties, receive feedback through observing consequences of their approaches, and iteratively adjust their strategies based on what they learn (Dornan et al., 2014). The computer science course created precisely these conditions—students attempted to apply memorization-based approaches, discovered they did not work through the immediate feedback of non-functioning code or debugging challenges, and were compelled to experiment with alternative strategies simply because they needed to complete assignments and had exhausted their usual approaches. This process was rarely comfortable or efficient; students described feeling their way forward through trial and error, often unsure whether new approaches would prove successful but willing to try them out of necessity. Yet these inefficient, uncertain experiments gradually accumulated into more substantial shifts in how students engaged with learning, including increased willingness to begin work before feeling fully prepared, greater comfort with iterative refinement through multiple attempts, and developing capacity to use errors as information rather than experiencing them as failures. The social dimension of this adaptation also proved important, with students describing how working alongside peers who were similarly struggling helped normalize difficulty and created opportunities for collaborative sense-making that individual study could not provide (Lee et al., 2024; Hägg-Martinell et al., 2015; Lave and Wenger, 1991). This finding suggests that learning environments designed to support productive academic transitions should not attempt to eliminate struggle or shield students from difficulty, but rather should create conditions where struggle is expected and normalized, where immediate feedback makes the consequences of different approaches visible, and where collaboration allows students to share strategies and collectively make sense of what works and why.

These findings carry significant implications for how public health curricula might be structured to better prepare students for the diverse learning demands they will encounter throughout their training. Traditional approaches to public health curriculum design often assume that students arrive with effective learning strategies and focus primarily on sequencing content logically and ensuring adequate time for mastery of epidemiological methods, health policy, and biostatistical frameworks, yet the accounts of the 10 students in this study suggest that at least some high-achieving students may enter public health programs with learning strategies that, while effective for memorization-based assessment, are comparatively narrow and less readily adapted to reasoning-intensive, ambiguous, or data-analytical contexts—an interpretation broadly consistent with prior work on the limited transfer of learning strategies and on preparedness for new learning demands (Peters et al., 2018; Surmon et al., 2016), though one that must be generalized cautiously given the small, self-selected sample. By intentionally incorporating academic transitions that challenge students' established approaches early in their education, curricula could help students develop more adaptive learning capabilities before they encounter high-stakes fieldwork placements and applied public health practice contexts where the consequences of inflexible strategies are more serious (Surmon et al., 2016; Tolsgaard et al., 2020; Lim et al., 2024a; Mirbahai et al., 2024). This does not mean introducing difficulty arbitrarily or overwhelming students with demands they cannot meet, but rather thoughtfully designing experiences where familiar strategies prove insufficient, where students must develop alternative approaches through supported experimentation, and where reflection helps students articulate what they are learning about learning itself. Such experiences might include not only courses from outside traditional public health sciences, as in this study, but also problem-based learning scenarios involving real-world health data that resist template solutions, case analyses requiring synthesis of evidence across conflicting epidemiological studies, or collaborative projects demanding negotiation and collective reasoning about population health priorities (Lutz et al., 2025; Pham et al., 2023; Humphries et al., 2022). The key is creating conditions where transfer of familiar strategies fails in psychologically safe contexts, prompting students to consciously examine their assumptions about learning and develop more varied approaches they can deploy flexibly as contexts demand (Chaffey et al., 2012; Mann et al., 2009). Ultimately, helping students navigate productive academic transitions may prove as important as teaching them specific public health content, as it equips them with the adaptive capacity needed for lifelong learning in a profession where evidence evolves rapidly, data systems grow increasingly complex, and practice contexts shift with emerging health challenges. In practical terms, the findings point to concrete initiatives that programme directors could pilot and evaluate in future iterations of the curriculum: an early, low-stakes ‘learning-to-learn' component; scaffolded programming or data tasks that deliberately normalize iteration and error; and structured reflection prompts that help students name and adapt their study strategies before higher-stakes courses and fieldwork.

## Strengths and limitations

This study has several strengths and limitations that should be weighed when interpreting its findings. Its principal strength lies in the rich, in-depth qualitative account it provides of how students experience and make sense of an unfamiliar learning transition in an authentic, practice-based course, with interviews timed mid-course so that experiences were captured while still vivid. At the same time, the study is best understood as a small-scale, exploratory, or pilot study.

The sample comprised only 10 students who volunteered to take part, and this self-selection means the findings may over-represent students who remained engaged and willing to reflect on difficulty, introducing a selection bias that limits how far the conclusions can be generalized. Data were also collected at a single, mid-course time point; a before-and-after design comparing students' accounts early and late in the course would offer a more balanced and developmental picture of adaptation and would more directly inform interventions pitched at the point of entry into such courses. Accordingly, the findings are offered as transferable insights and hypotheses for further study rather than as statistically generalizable claims; they could usefully be extended to other courses within the same programme, and to comparable programmes, through larger and longitudinal designs. Relatedly, much of the transition and preparedness literature drawn on in this paper originates in adjacent health-professions education, particularly medical education; its relevance to public health students is plausible but not firmly established, and the comparisons offered here should be read with that boundary in mind.

## Conclusion

This study explored how first-year public health students experience academic transition when encountering a computer science course that challenges their established learning strategies. The findings suggest that students entered the course with deeply ingrained expectations about how learning should work, shaped by years of success achieved primarily through memorization and careful reproduction of demonstrated procedures. When these familiar approaches proved inadequate for programming tasks that demanded logical reasoning, iterative problem-solving, and tolerance for ambiguity, students experienced not simply difficulty with new content but a fundamental disruption of their assumptions about learning itself. This academic transition shock prompted gradual adaptive responses as students experimented with unfamiliar approaches out of necessity, developing increased willingness to engage with uncertainty, learning to use errors as information rather than experiencing them as failures, and beginning to reconceptualize academic competence as involving reasoning and persistence rather than recall alone. These shifts emerged not from explicit instruction but through iterative engagement with tasks that resisted familiar strategies, suggesting that productive academic transitions may serve important developmental functions by exposing limitations in students' existing approaches and creating conditions where adaptation becomes necessary.

The implications of these findings extend to the broader design of public health curricula and the intentional use of academic transitions as developmental opportunities. If many students enter public health programs with effective but narrow learning strategies that prove inadequate when demands shift, then curricula should thoughtfully incorporate experiences that challenge these strategies early in training, creating supportive contexts where students can discover limitations in their approaches and develop more flexible learning capabilities before encountering high-stakes fieldwork and applied practice environments. Future research should examine how public health students' experiences of academic transition in structured coursework contexts like computer science courses influence their capacity to navigate subsequent transitions into fieldwork placements and professional practice, and whether early exposure to learning demands that resist memorization-based approaches better prepares students for the analytical and adaptive challenges of public health work. Investigating individual variation in how students respond to academic transition shock, identifying factors that facilitate productive adaptation versus disengagement, and exploring pedagogical approaches that best support students through these transitions would strengthen understanding of how to design public health curricula that develop not just disciplinary knowledge but the adaptive learning capabilities essential for lifelong professional growth in a rapidly evolving field.

## Data Availability

The raw data supporting the conclusions of this article will be made available by the authors, without undue reservation.

## Appendix A. Semi-structured interview guide

The semi-structured interview guide below provided the framework for each interview. As is usual in semi-structured interviewing, the wording and order of questions varied between interviews, and follow-up probes were used to explore participants' responses in depth.

1. Opening / expectations: Before this course began, what did you expect it would be like, and how did you expect to study for it?

2. How has your actual experience of the course compared with those expectations?

3. Difficulty moments: Can you describe a specific moment when you felt confused, frustrated, or surprised by what the course demanded?

4. Strategy failure: When your usual ways of studying did not seem to work, what did you do?

5. Comparison: What did you find most difficult about learning programming compared with your other subjects?

6. How did this course compare with your other public health courses? Did the difficulty feel qualitatively different from typical academic difficulty?

7. Change over time: Have you noticed any changes in how you approach learning as the course has progressed?

8. Emotional response: How did you feel when you got stuck, and did that change over time?

9. Closing: Is there anything else about your experience of this course that you would like to add?
